# The impact of employment on mental healthcare use among people with disability: distinguishing between part- and full-time employment

**DOI:** 10.5271/sjweh.4123

**Published:** 2023-11-01

**Authors:** Karinna Saxby, Helen Dickinson, Dennis Petrie, Anne Kavanagh, Zoe Aitken

**Affiliations:** 1 Centre for Health Economics, Monash Business School, Monash University, Building H, 900 Dandenong Road Caulfield East, Victoria, Australia.; 2 The Melbourne Institute of Applied Economic and Social Research, University of Melbourne, 111 Barry Street, Carlton, Victoria, Australia.; 3 School of Business, UNSW Canberra, Australian Capital Territory, Australia.; 4 Centre for Health Policy Melbourne School of Population and Global Health, The University of Melbourne, Melbourne, Victoria, Australia.

**Keywords:** health inequality, mental health, PWD, people with disability, persons with disabilities

## Abstract

**Objective:**

Employment can improve mental health among people with disability (PWD), however, little is known about how different levels of workforce participation influence mental healthcare use. The aim of this study was to estimate the extent to which different levels of working hours are associated with changes in mental healthcare use among PWD.

**Methods:**

Data on working hours and healthcare use among working age PWD who were receiving government benefits (N=260 825) was obtained from Australian Census-linked administrative records between 2011 and 2019. Individual fixed effects panel models were used to estimate the impact of increased working hours on mental healthcare (services and prescriptions). Heterogeneity analyses by job security and key sociodemographic characteristics were conducted.

**Results:**

Compared to not working, we found that working 1–14, 15–29, and ≥30 hours per week was respectively associated with a 3.3%, 18.0%, and 9.9% reduction in the use of mental healthcare prescriptions as well as a 6.8%, 18.4%, and 22.3% reduction in the use of mental healthcare services by PWD. The effects were larger for PWD in more secure work and those living in rural and disadvantaged areas.

**Conclusions:**

Working more hours was associated with reduced mental healthcare use among PWD. Policy interventions should consider the broader benefits of enabling part-time and secure work placements for PWD, particularly for those living in rural and disadvantaged regions.

Approximately 15% of the world’s population has a disability (1). There are variations in definitions across the literature, however disability is broadly defined as an impairment, limitation, or condition which restricts everyday activities and has lasted, or is likely to last, for at least six months (1, 2). People with disability (PWD) experience poorer mental health and use higher levels of inpatient, ambulatory, and pharmacological mental healthcare than the general population (3–7). Understanding factors that improve mental health and reduce associated mental healthcare costs among PWD remains a critical policy issue.

PWD disproportionately experience social exclusion, economic disadvantage, and unemployment (8, 9), therefore enhancing workforce participation is seen as an important lever for improving social inclusion and economic empowerment among PWD. Employment is also likely to have beneficial impacts on mental health (10), particularly given unemployment and material conditions (for example, economic deprivation) has been shown to contribute to disability-related mental health inequalities for people without disability) (11–13). Indeed, several studies indicate that employment is associated with improved mental health among the general population and that these effects are more pronounced for PWD (10, 14–18).

However, little is known about how different levels of workforce participation influences mental health or mental healthcare use among PWD. Disentangling the effects of different levels of workforce participation is particularly important as some PWD may not have the capacity to work full-time or may only be able to find part-time work. Indeed, in Australia, approximately 41% of employed working-age PWD work part-time (2). Moreover, PWD are more likely to be in insecure work (19, 20) and reside in more rural and socioeconomically deprived regions (21), so an understanding of the heterogeneous effects of employment by degree of job security and regionality for PWD is a crucial, yet underexplored, research question.

This knowledge gap is partly due to data limitations and methodological challenges (22–24). For example, relatively small sample sizes within population level datasets restrict subgroup analyses (22). Understanding the magnitude and direction of the impact of employment on outcomes is further complicated by challenges associated with reverse causality (for example, mental health affecting work and vice versa) as well as unobservable individual factors that could be correlated with both employment and mental health (for example, level of impairment, familial factors) (24–26). Consequently, the effects of employment are likely to be biased when not accounting for unobserved confounding (25).

This study addresses these limitations, using newly available whole-of-population administrative data to estimate the extent to which different levels of working hours are associated with changes in mental healthcare use among Australian PWD. We additionally contribute to the evidence base by exploring whether effects vary by job security and other key sociodemographic characteristics.

## Methods

### Data

The data sourced for this analysis comes from the Multi Agency Data Integration Project (MADIP), an individual-level linked dataset of the 2011 Australian Census of Population and Housing (the Census) combined with data on demographics, medical care, employment, education, government payments, income and taxation (27–29). The sample for analysis was constructed using data from the Census, government payments, and medical care provided under Australia’s universal health insurance scheme: Medicare.

The population of PWD for this analysis was defined as those who reported a ‘core activity need for assistance’ in the Census. The Australian Bureau of Statistics (ABS) developed the core activity need for assistance variable to measure profound or severe disability, which is defined as “those needing assistance in their day to day lives in one or more of the three core activity areas of self-care, mobility and communication because of: a long-term health condition (lasting six months or more); a disability (lasting six months or more); or old age” (30). People who report limitations in any one of the three core activities and always or sometimes require assistance in these areas are classified as having profound or severe disability (30). The ABS provides an aggregated variable of core activity need for assistance that is derived from these questions. As such, the sample represented people with severe or profound disability, representing approximately 6.5% of the 2011 Census population and 3.4% of the working age population (aged 15–64 years) (30).

Depending on individual circumstances, PWD who meet the medical and non-medical eligibility criteria can receive means-tested income support through either the disability support pension (DSP) or ‘jobseeker’ (previously known as ‘new start’) allowance. DSP is available for those who have reduced capacity to work because of their disability and are <65 years old (at which point they would have qualified for the age pension). In 2020, the most common primary medical conditions of working-age DSP recipients were psychological or psychiatric conditions (37%), followed by musculoskeletal conditions (18%), and intellectual or learning conditions (17%) (31). Jobseeker allowance is more targeted to individuals who are unemployed. However, in 2020, roughly 25% of jobseeker allowance recipients were assessed as having partial capacity to work (defined as being able to work<30 hours a week) (31).

The government payments dataset in MADIP (provided via Department of Social Services Data Over Multiple Individual Occurrences, ‘DOMINO’) provides information on payment type and duration (with start and end dates for each payment) for all individuals from 2006 through until the end of 2019. When individuals are receiving DSP/jobseeker allowance, the DOMINO dataset reports information on the number of hours worked. To this end, we can only observe hours worked while individuals are receiving income support payments.

All Australian citizens and permanent residents have access to free or subsidized healthcare under Australia’s universal health insurance scheme Medicare. In MADIP, the Medicare Consumer Directory contains all Medicare customer records from January 2011 to December 2020. Medicare records contain information on out-of-hospital and private hospital medical services through the Medicare Benefits Schedule (MBS) and information on prescription medicines through the Pharmaceutical Benefits Scheme (PBS). As use of outpatient care, public hospital care, and private prescriptions are not available, we restricted analysis to out-of-hospital services and prescription medicines, following previous analyses of Medicare records (27, 32).

**Figure 1:**
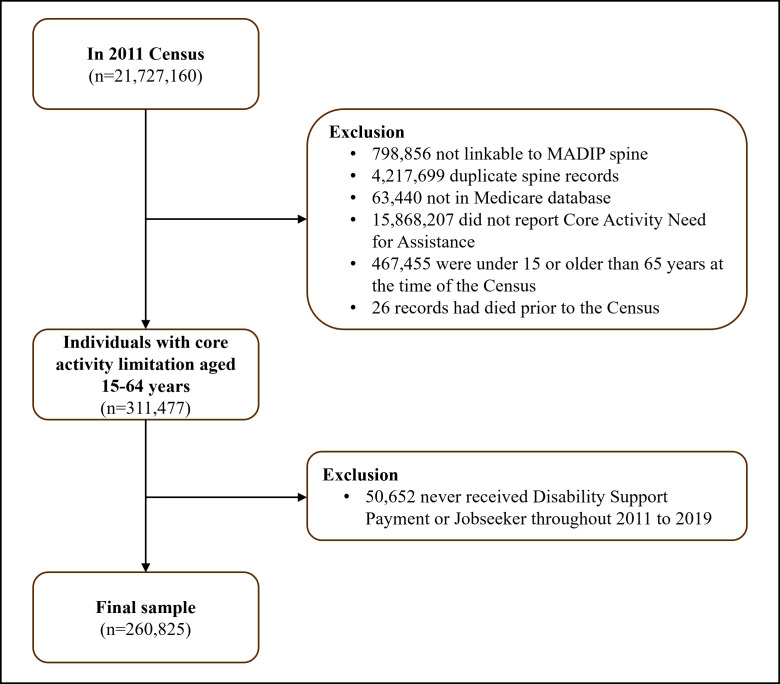
Flowchart illustrating the identification of the study population.

The baseline sample was derived from all individual records in the 2011 Census that were linkable and not duplicated in MADIP (33). The MADIP sample has been shown to be representative of the Census population (27). We then excluded individuals who were not linked to the Medicare database as we were unable to view healthcare use for these individuals (this includes, for example, temporary and overseas visitors without permanent residency). We then restricted the sample to individuals who reported a core activity limitation, were 15–64 years at the time of the Census, and had not died prior to the Census. Restriction to the ‘traditionally working age’ population reduces potential bias from including older individuals who may be retired or report needing assistance with core activities due to old age. Finally, we restricted the sample to those who had received either DSP/jobseeker allowance at any time between 2011 and 2019. The majority of the working age sample who reported a core activity limitation had received DSP/jobseeker allowance throughout 2011 and 2019 (84%). This resulted in a final sample 260 825 individuals (figure 1). Individuals who died or turned 65 years old throughout the observation window were censored at that time point.

### Outcomes

Two outcome variables were explored: utilization of mental healthcare services and mental health prescription medicines. These details are provided through the MBS and PBS records, respectively. Mental healthcare service use included all mental health services reimbursed by the government, as identified by the Australian Institute of Health and Welfare (a full list is provided in the supplementary material, www.sjweh.fi/article/4123, table S1) (34). We included all government reimbursed mental healthcare as we wanted to understand whether transitions into part- or full-time work could offset government contributions toward mental healthcare. While some activities may not directly involve the patient (for example, healthcare professionals’ collaboration with formal and informal caregivers), these indirect activities still play a critical role in the overall management and coordination of patients’ mental healthcare, and are subsidized by the government. Nevertheless, we also considered different categories of mental healthcare services. Namely, those services provided by general practitioners (GP), psychologists, psychiatrists, and allied health providers. Mental health prescriptions were defined as use of antidepressants and anxiolytics, identified in the PBS dataset using Anatomical Therapeutic Chemical (ATC) codes N05B and N06A respectively. This specification was applied because antidepressants and anxiolytics are the most commonly prescribed for mild-to-moderate depression and general anxiety (35–37). Benzodiazepines listed as ‘hypnotics and sedatives’ (N05C) were not included as these are also indicated for other non-mental health conditions which could confound the analysis, such as insomnia or epilepsy (38).

MBS and PBS records in MADIP ran from January 2011 to December 2020 and provided exact dates for each service received and prescription dispensed. For each individual, we summed the total number of mental healthcare services used (MBS items as per supplementary table S1) and prescription medicines (antidepressants/anxiolytics) filled per quarter between 2011 and 2019. Data from 2020 onwards was excluded due to the COVID pandemic and its potential to confound both exposure and outcome variables.

### Exposure

The key explanatory variable of interest was weekly working hours constructed from the DOMINO data using the variable on “the total working hours worked per fortnight” reported in all jobs. The number of hours worked per week was then averaged for each individual in each quarter, from 2011 to 2019, and categorized as 0, 1–14, 15–29, and ≥30 hours. People who worked on average 0 hours per week in a quarter were considered to be not working (either unemployed or not in the labor force), those working 1–14 or 15–29 hours per week were considered to be employed part-time, and those working ≥30 hours per week on average were considered to be in full-time employment. Grouping to ≥30 hours was selected as it aligns with Australian government classifications of “partial capacity to work” being <30 hours, and OECD classifications (31, 39).

### Potential confounders

There are multiple confounders that could have affected both transitions into employment and utilization of mental healthcare. These included age, sex, residential location and healthcare accessibility, education, country of birth, family background, household structure, severity of disability, socioeconomic factors, and individual preferences (27, 40–42). We therefore controlled for individual fixed effects. This implicitly controlled for both observable and unobservable time-invariant individual characteristics because they do not vary within individuals. Additionally, we included age-bin fixed effects (15–25, 25–44, and 45–64 years) and calendar year as covariates in the models. This enabled us to partially control for within-individual time varying factors as well as external factors that might have influenced use of mental healthcare among PWD over time, such as changes to government remuneration of mental healthcare and the items subsidized.

### Analysis

Our data captures repeated measures of both exposures (working hours) and outcomes (mental healthcare use) for the same individuals over time. We used longitudinal linear fixed-effects regression analyses to estimate the effect of changing working hours on the use of mental healthcare within individuals. In this approach, each individual serves as their own control, and we use a mean-centring approach to model how within-person variation in exposures from the individuals’ mean value is associated with deviations in the outcomes of interest from the individual’s mean value (18, 42). Using unemployed (working 0 hours per week) as the reference category, coefficients generated from these models therefore describe the average change in use of mental healthcare associated with different levels of working hours compared with when each individual was unemployed (working 0 hours per week). Since this is a within-person analysis, this approach estimates the changes in mental healthcare associated with transitioning between different categories of working hours for each individual, which are averaged across the sample to estimate average effects. For example, if an individual transitioned between working 0 hours to working 1–14 hours per week, the within-person change in mental healthcare use would be estimated as the difference between their mean mental healthcare use in all observations in which they worked 1–14 hours per week and mean mental healthcare use in observations when they were unemployed. It is important to note that estimates are derived from changes in working hours in both directions, such that the estimates of changes in mental healthcare use are derived from effects of either losing or gaining work. However, the coefficients are interpreted as the effect of gaining employment. To avoid challenges with reverse causality in longitudinal data (24, 43), we quantified the effect of working hours on use of mental healthcare services and mental health prescriptions in the following quarter. This ensured that the exposure preceded the outcome. We selected this timeframe for our baseline model as PWD are more likely to work in precarious and short-term employment (19). In addition, for sensitivity analyses, we explored whether the effects vary over longer time frames by estimating whether working hours are associated with mental healthcare use in the following three quarters (that is, for up to a year after exposure).

In further heterogeneity analysis, we explored whether associations between increased working hours and mental healthcare use varied by key demographic subgroups. This was achieved by including an interaction term between the exposure and each demographic variable in turn and presenting stratum-specific estimates for each category of the demographic variables. Specifically, we estimated whether there was variation by age group (15–25, 25–44, 45–64 years), sex (male, female), rurality [urban, rural (inner regional, outer regional, and remote) as defined by the Australian Government (44)], area-level disadvantage (above/below median) (45), and education (high school or less, university level). More stable employment has been associated with improved mental health outcomes (46), so we additionally examined whether heterogeneous effects exist for individuals in more secure employment; specifically, focusing on those who have reported positive working hours for both the current and previous quarter. Hypothesis testing for subgroup analyses was achieved by comparing confidence intervals (47). All analyses were conducted using STATA version 17, StataCorp, College Station, TX, USA.

## Results

### Descriptive statistics

Table 1 describes the characteristics of the analytic sample. The mean age was 46 years, with 61% of the sample being aged ≥45 years at the time of the 2011 Census. Receipt of DSP throughout the observation window was much higher relative to the jobseeker allowance (93% versus 12%). The majority of the sample (64%) had high school or less education, lived in major cities (64%), and lived in areas with higher than median levels of socioeconomic disadvantage (61%). In the first observed quarter, the majority (80%) were not working. Over the entire observation window, individuals worked on average 1.3 hours per week. Among those who reported positive working hours, individuals worked on average 10.6 hours per week. Individuals in the sample used on average 0.10 mental healthcare services and 0.52 mental health prescriptions each quarter. Disaggregation by care type showed that, each quarter, individuals used on average 0.04 psychiatrist services, 0.03 GP-related mental healthcare services, 0.03 psychologist services, and 0.003 allied health mental health services. Within mental health prescriptions, use of antidepressants was higher than anxiolytics (0.37 and 0.15 scripts per quarter respectively).

**Table 1:** Descriptive characteristics for those reporting a 'core activity need for assistance' and ever received disability support payment / jobseeker allowance between 2011–2019 (N=260 825) [IRSD=index of relative socioeconomic disadvantage].

		Mean	%	Frequency
Age ^a^		45.74		
Age group ^a^				
	<25		12	32 234
	25–45		27	70 236
	>45		61	158 355
Highest educational attainment ^a^				
	High school or less		64	166 637
	Diploma or certificate		17	43 937
	University degree		6	14 529
Living in major city ^a^			64	167 450
Living in above median IRSD ^a, b^			39	102 970
Ever disability support payment ^c^			93	243 082
Ever jobseeker ^b^			12	30 881
Died ^b^			12	32 529
Ever reported hours worked (including 0 hours) ^c^			92	238 685
Ever reporting positive hours worked ^c^			22	57 991
Ever reporting positive hours in continuous employment ^c^			8	20 930
Ever reporting positive hours in variable employment ^c^			20	52 206
Average no. quarters on payment ^c^		37.24		
Average no. quarters reported positive hours ^c^		11.62		
Working hours ^c^				
	Unemployed (0 hours)		80	209 656
	0–15		4	11 401
	15–30		2	4999
	>30		1	2904
Hours worked per week ^c^		1.29		
Hours worked per week for positive hours ^c^		10.63		
No. mental health services per quarter ^c^		0.10		
No. mental health related GP services per quarter ^c^		0.03		
No. psychologist services per quarter (2011–2019)		0.03		
Mental health related allied health services per quarter ^c^		2.69E-03		
No. psychiatrist services per quarter ^c^		0.04		
No. mental health scripts per quarter ^c^		0.52		
No. antidepressant scripts per quarter ^c^		0.37		
No. anxiolytic scripts per quarter ^c^		0.15		

^a^ Based on responses in 2011 census. ^b^ Higher scores equate to regions with ‘less disadvantage’ ^c^ Throughout observation window 2011 to 2019. ^d^ Based on values observed in first quarter when receiving DSP/Jobseeker payment.

### Regression analyses

The results of the fixed-effects regression models for mental healthcare use are presented in table 2. These results show, on average, how healthcare use changes when people worked 1–14, 15–29, or ≥30 hours per week compared to when they worked 0 hours per week. Compared to not working, working 1–14 hours was associated with a 6.8% and 3.3% reduction in the use of mental healthcare services and mental health prescriptions respectively. Working 15–29 or ≥30 hours per week was also associated with larger reductions in the use of mental healthcare however the magnitude of the effect size was similar for working 15–29 or ≥30 hours for both outcomes. Compared to not working, working 15–29 or ≥30 hours was associated with a 18.4% and a 22.3% reduction in mental healthcare use respectively as well as an 8.0% and 9.9% reduction in mental health scripts respectively. The full regression analyses exploring the effects over longer time frames are presented in supplementary table S2. These results show that, compared to the baseline model, working hours is associated with slightly smaller reductions in mental healthcare service use in the second quarter after exposure. The longer-term associations appear more apparent for mental health prescriptions. Although effects are again slightly smaller relative to the baseline model, working more hours is significantly associated with reduced use of mental health scripts for up to one year (four quarters) after exposure.

**Table 2:** Estimated mean difference in mental healthcare use per quarter for categories of working hours compared to not working (number person-quarter observations=6 669 765). Standard errors are robust clustered at the individual level. Means provided for each outcome at the quarterly level (overall mean for the whole analytic sample across all quarters). All models control for individual effects, age bin, and year fixed effects.

Working hours per week	Mental health services used in following quarter (Mean of outcome=0.103)				Mental health scripts used in following quarter (Mean of outcome=0.523)		
	ß	95% CI	% change of ß relative to mean		ß	95% CI	% change of ß relative to mean
Not working (0)	Ref	Ref	Ref		Ref	Ref	Ref
1–14	-0.007 ^a^	-0.011– -0.003	-6.8		-0.017 ^a^	-0.022– -0.012	-3.3
15–29	-0.019 ^a^	-0.024– -0.014	-18.4		-0.042 ^a^	-0.049– -0.035	-8.0
≥30 or more	-0.023 ^a^	-0.029– -0.017	-22.3		-0.052 ^a^	-0.061– -0.042	-9.9

^a^ Significant at 1%.

**Figure 2:**
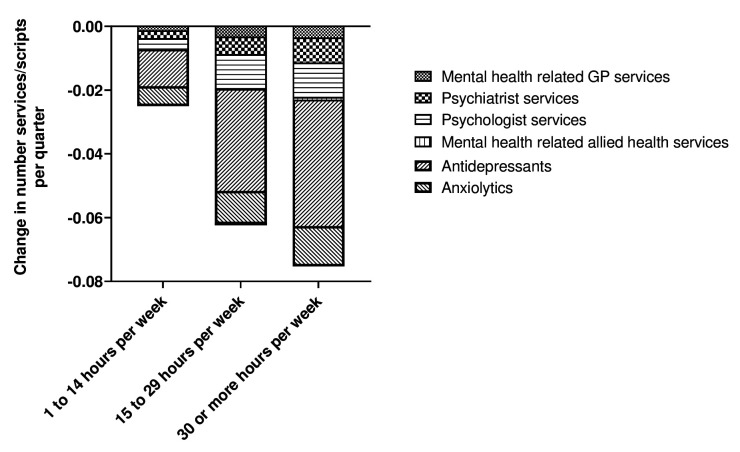
Change in mental healthcare use by category of care.

The breakdown of the change in types of mental healthcare used are presented in figure 2. This indicates that the largest reduction in mental healthcare services associated with working more hours was mainly attributed to fewer psychologist services, followed by psychiatrist services, and mental health-related GP services. Similarly, the largest reduction in mental healthcare prescriptions associated with working more hours was mainly attributed to reduced use of antidepressants, followed by anxiolytics.

**Figure 3:**
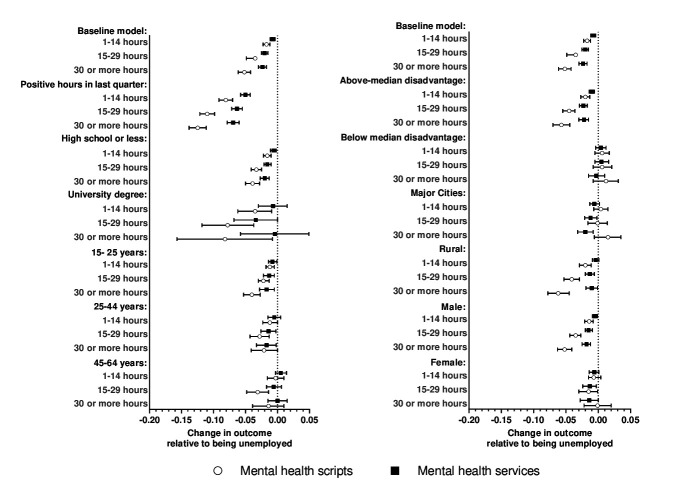
Heterogeneity analysis by job security, educational attainment, age group, rurality, regional disadvantage, and sex. Notes: Point estimates represent estimated mean differences in use of mental health scripts/services per quarter for categories of working hours compared to not working. Average results for the baseline sample presented at the top of each panel to facilitate relative interpretation of coefficients among different subgroups.

The heterogeneity analyses, presented in figure 3 show how the association between working hours and the use of mental health services and mental health scripts varies by age group, education, rurality, area-level disadvantage, job security, and sex. Patterns in use of mental healthcare services and prescriptions were broadly similar across different subgroups. Compared to the baseline model, there was generally no evidence of heterogeneity in the effects of working hours on use of mental healthcare services and prescriptions by level of education, age group, and sex. However, the associations were less precisely estimated for those with university degrees, those >45 years old, and females. This suggests that the observed relationships were generally attributed to younger populations, those with high school or less education, and males. The associations between hours worked and mental healthcare use were larger for PWD living in areas with higher levels of disadvantage and more rural areas; with little to no effects observed for those living in more advantaged areas. There were also significantly larger effects for PWD in more secure employment; with effect sizes almost doubling if individuals had reported positive working hours for the past two quarters.

## Discussion

The purpose of this study was to investigate the association between working hours and use of mental healthcare among PWD.

Increased working hours were consistently associated with a reduction in the use of both mental healthcare services and scripts among PWD. Importantly, the findings show that the magnitude of the reduction in the use of mental healthcare services and scripts was similar for PWD who worked part time (15–29 hours per week) and full time (≥30 hours per week) and that effects were more pronounced for PWD living in rural and disadvantaged areas as well as those who had been in more secure employment.

These findings align with previous studies which have documented higher use of healthcare among unemployed compared to employed in the general population (48, 49) as well as for PWD on income support (50). Our results also align with another study in the general population which found that those employed in secure work used fewer antidepressants than those in more insecure work (46). To the extent we can equate demand for mental healthcare with need, these results also align with previous research which has indicated that employment had a positive impact on self-reported mental health among PWD (11–14, 18) and, more specifically, that secure work is associated with better mental health (46, 51).

There are several reasons we would expect to see these associations with employment and mental healthcare use among PWD. Consistent with a large body of empirical evidence (11–14, 18), it is possible that reductions in mental healthcare are due to reduced need for care (that is, improved mental health). Previous research also suggests that these effects are largely driven by improvements in income and social support (51, 52). However, as we are estimating an average effect (ie, from either reducing or increasing hours) it is also possible that changes in working hours could reduce individuals’ capacity to seek care. For example, if working hours reduce, individuals may be spending more of their time seeking a new role or be less able to afford out-of-pocket mental health expenses. Although, given the effects are relatively similar for those who worked part time (15–29 hours per week) and full time (≥30 hours per week), this mechanism may be less plausible.

The results of this study should also be interpreted within the context of several limitations. First, on aggregate, the results suggest that increased working hours improves mental health and subsequently reduces mental healthcare use, however it is possible that this might not be the case for all PWD. As only an aggregated variable of core activity need for assistance is provided, we cannot distinguish between different types of disability (eg, intellectual, physical, or functional limitation). Moreover, our sample was restricted to people with severe or profound disability receiving income support payments. To this end, we were not able to observe how transitions in and out of work impact the broader population of PWD who are not receiving benefits. This could include PWD in permanent employment and those who were not at least partially attached to the welfare payment system, such as higher income households. However, given increased working hours were not significantly associated with changes in mental healthcare for those in more affluent regions, we may expect that the impact of working hours would be smaller for higher income groups. It is possible that the effect of working hours on mental healthcare could also differ for people with mild and moderate disability or different disability groups. Future research should consider these important subgroups. In particular, recent additions to the 2021 Census which posed questions on different types of long-term health conditions (53) as well as the core activity need for assistance module, could be considered as data collections mature. Nevertheless, these results capture an important, and majority, subgroup of those with severe or profound core activity limitations. This subgroup has also historically been taken as a priority population for Australia’s specialist disability services and government policy (54).

Second, as the MADIP does not currently contain information on inpatient or emergency department services, we were unable to explore whether more working hours were associated with reductions in more acute types of mental healthcare. If data integration in this space improves, this could be a key avenue of future research. Finally, the coefficients representing the effects on mental healthcare use associated with changes in working hours generated from the fixed-effects model are derived from changes in working hours in both directions, thus averaging the effect of both losing and gaining work. It is possible that the positive effects of working, such as building social connections, may take longer to manifest than the negative effects of losing work. This would also align with our results showing that reductions in mental healthcare were more pronounced among those working in more secure employment. Alternatively, as aforementioned, increased working hours could hamper individuals’ ability to seek care. Future research should therefore aim to disentangle the dynamics of positive and negative changes in employment.

Despite these limitations, our study offers distinct advantages and is the first to provide evidence on how both part- and full-time work influence mental healthcare use among PWD. Linked whole-population data over nine years provided representative and robust evidence for not only PWD receiving benefits but also for previously understudied subgroups within this population. The nature of our individual fixed-effect analysis also enables us to control for bias associated with time-invariant confounding and reverse causality. These results have clear policy implications. In addition to the potential benefits associated with improved mental health, the reductions in mental healthcare infer that there are wider benefits to the economy, through reduced healthcare costs. Socioeconomically disadvantaged populations disproportionately experience poor mental health (55, 56), therefore the larger reductions in mental healthcare use observed among PWD in disadvantaged areas could present a particular benefit in terms of closing the gap in mental health inequalities. There is an extensive policy focus on financial incentives to encourage workforce participation among beneficiary recipients (57–60) and the results presented herein deliver key insights for policy design in this priority population. For instance, showing that part-time work reduces mental healthcare to a similar magnitude as full-time work suggests that policies which strengthen incentives for part-time and secure work placements for PWD are necessary. These could include allowances such as enhancing workplace flexibility, through social or built environment accommodations (61–63) or other direct interventions such as employee assistance programs and focused psychological strategies (64–66). Indeed, in the US and Scandinavian countries, such measures have led to reductions in government social costs and enhanced work participation among both the general population and PWD (67–70).

These findings are particularly relevant to the Australian context where current government programs, such as Disability Employment Services, have stronger incentives for positioning PWD into full- rather than part-time employment. The Australian Government’s disability employment strategy also has a stronger focus on PWD with high job capacity (18, 71). Our results suggest that greater emphasis should be placed on enabling part-time and secure work placements for PWD. The potential for such programs to reduce mental health inequalities among PWD and reduce government mental healthcare costs likely warrants ongoing government investment.

## Supplementary material

Supplementary material

## Data Availability

Data is available upon application from the Australian Bureau of Statistics.

## Protection of research participants

Findings based on the use of deidentified administrative microdata. The information that the ABS collects from individuals and agencies for the MADIP is covered by the secrecy provisions of the Census and Statistics Act 1905. Privacy Impact Assessments (PIAs) are an important part of the governance and accountability framework for MADIP. PIAs help identify and manage the privacy impacts of MADIP. The ABS is trusted as the Accredited Integrating Authority for MADIP.
