# Supplementary material for: The impact of employment on mental healthcare use among people with disability: distinguishing between part- and full-time employment
Source: Scand J Work Environ Health. 2023 Oct 31;49(8):598–609. doi: 10.5271/sjweh.4123 (PMC11402066; doi:10.5271/sjweh.4123)
Supplement: Supplementary material [file SJWEH-49-598-S001.pdf]

The impact of employment on mental healthcare use among people with disability: distinguishing between part- and full-time employment[1]

by Karinna Saxby, PhD, [2] Helen Dickinson, PhD, Dennis Petrie, PhD, Anne Kavanagh, PhD, Zoe Aitken, PhD

1. Supplementary material
2. Correspondence to: Karinna Saxby, The Melbourne Institute of Applied Economic and Social Research, University of Melbourne, 111 Barry Street, Carlton, Victoria, Australia. [E-mail: karinna.saxby@unimelb.edu.au]

Table S1. Mental health services item numbers

The following list of Medicare-reimbursed mental health related care is provided directly from the Australian Institute of Health and Welfare (36). This is a complete list of all Medicare-subsidised mental health specific services that were reimbursed at any time on the Medicare Benefits Schedule (MBS) between 2011 to 2022. This complete list therefore includes all MBS item numbers including those that may have been discontinued or added, aggregated, or otherwise amended throughout this observation window. Not all item numbers will be present in our sample. For example, as our analysis runs from 2011 to 2019, COVID-19 related mental health services are not included. Similarly, we have excluded inpatient mental health services.

Provider type	Item group	MBS group & subgroup	MBS item numbers
**Psychiatrists**	**Initial consultation new patient**	Group A8	296, 297, 299
	**Patient attendances – consulting room**	Group A8	291, 293, 300, 302, 304, 306, 308, 310, 312, 314, 316, 318, 319
	**Patient attendances – consulting room- review of eating disorder treatment and management plan**	Group A36	90266
	**Patient attendances – videoconference - review of eating disorder treatment and management plan**	Group A36	90268
	**COVID-19 services – Patient attendances – telehealth**	Group A40	91827, 91828, 91829, 91830, 91831, 91837, 91838, 91839, 91840, 91841, 92461[(b)], 92462[(b)], 92463[(b)], 92464[(b)], 92465[(b)], 92501[(b)], 92502[(b)], 92503[(b)], 92504[(b)], 92505[(b)], 92506[(b)]
	**Patient attendances – hospital**	Group A8	320, 322, 324, 326, 328

Provider type	Item group	MBS group & subgroup	MBS item numbers
	**Patient attendances – other locations**	Group A8	330, 332, 334, 336, 338
	**Group psychotherapy**	Group A8	154[(a)], 155[(a)], 156[(a)], 342, 344, 346
	**COVID-19 services – Group psychotherapy**	Group A40	92455, 92456, 92457, 92495, 92496, 92497
	**Interview with non-patient**	Group A8	157[(a)], 158[(a)], 159[(a)], 348, 350, 352
	**COVID-19 services- Interview with non-patient**	Group A40	92458, 92459, 92460, 92498, 92499, 92500
	**Telepsychiatry**	Group A8	288, 353, 355, 356, 357, 358, 359, 361
	**Telepsychiatry follow up – face-to-face**	Group A8	364, 366, 367, 369, 370
	**Case conferencing**	Group A15	855, 857, 858, 861, 864, 866
	**Electroconvulsive therapy**	Group T1, Subgroup 13	14224, 340[(a)], 886[(a)]
	**Electroconvulsive therapy**	Group T3	153[(a)]
	**Referred consultation for assessment, diagnosis and development of a treatment and management plan for autism or any other pervasive developmental disorder (PDD)**	Group A8	289
	**Eating Disorders Treatment Plan**	Group A36	90260, 90262
	**COVID-19 services – Eating Disorders Treatment Plan**	Group A40	92162, 92166
	**COVID-19 services – telehealth and phone services**	Group A40	92172, 92178, 92434, 92435, 92436, 92437, 92466[(b)], 92474, 92475, 92476, 92477
**General practitioners**	**GP Mental Health Treatment Plan – accredited**	Group A20, Subgroup 1	2710[(a)], 2715, 2717
		Group A7	281, 282

Provider type	Item group	MBS group & subgroup	MBS item numbers
	**COVID-19 GP Mental Health Treatment Plan – accredited**	Group A40	92116, 92117, 92122, 92123, 92128, 92129, 92134, 92135
	**COVID-19 GP Mental Health Treatment Plan – accredited (aged care facility)**	Group A42	93402, 93406, 93407, 93410, 93411, 93433, 93434, 93437, 93438, 93441, 93442
	**GP Mental Health Treatment Plan – non-accredited**	Group A20, Subgroup 1	2700, 2701, 2702[(a)]
		Group A7	272, 276
	**GP Mental Health Treatment – other**	Group A20, Subgroup 1	2712, 2713, 2719[(a)]
		Group A7	277, 279, 894, 896, 898
		Group A30	2121, 2150, 2196
	**COVID-19 GP Mental Health Treatment Plan – non-accredited**	Group A40	92112, 92113, 92114, 92115, 92118, 92119, 92120, 92121, 92124, 92125, 92126, 92127, 92130, 92131, 92132, 92133, 92170, 92171, 92173, 92176, 92177, 92179
		Group A42	93421, 93422, 93423, 93451, 93452, 93453
	**COVID-19 GP Mental Health Treatment Plan – non-accredited (aged care facility)**	Group A42	93400, 93401,93403, 93404, 93405, 93408, 93409, 93431, 93432, 93435, 93436, 93439, 93440
	**COVID-19 GP Mental Health Treatment Plan – eating disorder services**	Group A36	90264, 90267, 90269, 90271, 90272, 90273, 90274, 90275, 90276, 90277, 90278, 90279, 90280, 90281, 90282
	**GP Eating Disorders Plan**	Group A36	90250, 90251, 90252, 90253, 90254, 90255, 90256, 90257, 90261, 90263
		Group A40	92163, 92167
	**COVID-19 GP Eating Disorder Treatment and Management Plan – Telehealth Service**	Group A40	92146, 92147, 92148, 92149, 92150, 92151, 92152, 92153, 92154, 92155, 92156, 92157, 92158, 92159, 92160, 92161

Provider type	Item group	MBS group & subgroup	MBS item numbers
	**Focussed Psychological Strategies**	Group A20, Subgroup 2	2721, 2723, 2725, 2727, 2729, 2731, 2733, 2735
		Group A7	283, 285, 286, 287, 371, 372, 941, 942
	**Focussed Psychological strategies – COVID-19 services**	Group A40	91818, 91819, 91820, 91821, 91842, 91843, 91844, 91845, 92182, 92184, 92186, 92188, 92194, 92196, 92198, 92200, 93287, 93288, 93291, 93292, 93300, 93301, 93302, 93303, 93304, 93305, 93306, 93307, 93308, 93309, 93310, 93311
	**Focussed Psychological Strategies - bushfire affected people**	Group A39	91283, 91285, 91286, 91287, 91371, 91372, 91721, 91723, 91725, 91727, 91729, 91731
	**Family Group Therapy**	Group A6	170, 171, 172, , 996(a), 997(a), 998(a)
	**Family Group Therapy**	Group A7	221, 222, 223
	**Electroconvulsive therapy**	Group T10	20104
	**3 Step Mental Health Process – general practitioner[(a)]**	Group A18, Subgroup 4	2574[(a)], 2575[(a)], 2577[(a)], 2578[(a)]
	**3 Step Mental Health Process – other medical professional[(a)]**	Group A19, Subgroup 4	2704[(a)], 2705[(a)], 2707[(a)], 2708[(a)]
**Clinical psychologists**	**Psychological Therapy Services**	Group M6	80000, 80001, 80005, 80010, 80011, 80015, 80020, 80021
	**Eating Disorders Psychological Treatment**	Group M16	82352, 82353, 82354, 82355, 82356, 82357, 82358, 82359
		Group M18	93076, 93079, 93110, 93113
	**Focussed Psychological Strategies - bushfire affected people**	Group M17	91000, 91001, 91005, 91010, 91011, 91015
	**COVID-19 Psychological Therapy Services**	Group M25	93312, 93313, 93330, 93331, 93332, 93333, 93334, 93335, 93375, 93376
	**Enhanced Primary Care**	Group M3	10968

Provider type	Item group	MBS group & subgroup	MBS item numbers
**Psychologists including clinical psychologists**	**Focussed Psychological Strategies (Allied Mental Health)**	Group M7	80100, 80101, 80105, 80110, 80111, 80115, 80120, 80121, 91169, 91170, 91183, 91184
	**Focussed Psychological Strategies - bushfire affected people**	Group M17	91100, 91101, 91105, 91110, 91111, 91115
	**COVID-19 Additional focussed psychological strategies**	Group M26	93316, 93319, 93350, 93351, 93352, 93353, 93354, 93355
	**Initial focussed psychological strategies**	Group M28	93381, 93382
	**COVID-19 Psychology health service – residential aged care facility**	Group M29	93512, 93535, 93557
	**Assessment and treatment of PDD**	Group A10	82000, 82015
	**COVID-19 Telehealth Services**	Group M18	91166, 91167, 91181, 91182, 93032, 93035, 93040, 93043
	**Allied health service for Indigenous Australians who have had a health check**	Group M11	81355
	**Eating Disorders Psychological Treatment**	Group M16	82360, 82361, 82362, 82363, 82364, 82365, 82366, 82367
		Group M18	93084, 93087, 93118, 93121
	**Initial services (allied health service for Indigenous Australians)**	Group M30	93590
**Other allied health providers**	**Enhanced Primary Care – mental health worker**	Group M3	10956
	**Focussed Psychological Strategies (Allied Mental Health) – occupational therapist**	Group M7	80125, 80126, 80130, 80135, 80136, 80140, 80145, 80146
	**Focussed Psychological Strategies (Allied Mental Health) – social worker**	Group M7	80150, 80151, 80155, 80160, 80161, 80165, 80170, 80171

Provider type	Item group	MBS group & subgroup	MBS item numbers
	**Focussed Psychological Strategies - bushfire affected people (occupational therapist)**	Group M17	91125, 91126, 91130, 91135, 91136, 91140
	**Focussed Psychological Strategies - bushfire affected people (social worker)**	Group M17	91150, 91151, 91155, 91160, 91161, 91165
	**Focussed Psychological Strategies - COVID-19 affected people**	Group M18	91172, 91173, 91175, 91176, 91185, 91186, 91187, 91188
	**COVID-19 Additional focussed psychological strategies**	Group M26	93322, 93323, 93326, 93327, 93362, 93363, 93364, 93365, 93366, 93367, 93356, 93357, 93358, 93359, 93360, 93361
	**COVID-19 Initial focussed psychological strategies**	Group M28	93383, 93384, 93385, 93386
	**Initial allied health service – mental health worker**	Group M29	93506, 93529
	**Initial allied health service for Indigenous Australians – mental health worker**	Group M30	93551, 93584
	**Follow-up allied health services for Indigenous Australians – mental health worker**	Group M11	81325
	**Eating Disorders Services**	Group M16	82350, 82351, 82368, 82369, 82370, 82371, 82372, 82373, 82374, 82375, 82376, 82377, 82378, 82379, 82380, 82381, 82382, 82383
		Group M18	93074, 93092, 93095, 93100, 93103, 93108, 93126, 93129, 93134, 93137

(a) Item discontinued.

(b) Item introduced in September 2021.

(c) Item numbers have differing implementation and discontinuation dates. Details are available in the Medicare Benefits Schedule books.

Table S2. Long term effects

Estimated mean difference in mental healthcare use per quarter for up to four quarters after exposure for categories of working hours compared to not working

**Outcome**	**Mental healthcare services per quarter**	**Mental healthcare services per quarter**	**Mental healthcare services per quarter**	**Mental healthcare services per quarter**
Time period	First quarter after exposure<br>(1)<br>β [95% CI]	First quarter after exposure<br>(2)<br>β [95% CI]	Third quarter after exposure<br>(3)<br>β [95% CI]	Fourth quarter after exposure<br>(4)<br>β [95% CI]
unemployed (0 hours)	0.00000	0.00000	0.00000	0.00000
	[0.00000,0.00000]	[0.00000,0.00000]	[0.00000,0.00000]	[0.00000,0.00000]
(0-15)	-0.00773***	-0.00584**	-0.00046	-0.00062
	[-0.01153,-0.00392]	[-0.00947,-0.00221]	[-0.00450,0.00357]	[-0.00492,0.00369]
[15-30)	-0.02016***	-0.00682**	-0.00654*	-0.00649*
	[-0.02519,-0.01512]	[-0.01161,-0.00202]	[-0.01216,-0.00092]	[-0.01241,-0.00056]
30 +	-0.02351***	-0.00540	-0.00431	-0.01082**
	[-0.02970,-0.01733]	[-0.01174,0.00094]	[-0.01129,0.00268]	[-0.01833,-0.00331]
No. person-quarter observations	*6,669,765*	*6,495,245*	*6,320,426*	*6,145,914*

Notes: * Significant at 10%. ** Significant at 5%. * Significant at 1%. Standard errors are robust clustered at the individual level. Means provided for each outcome at the quarterly level (overall mean for the whole analytic sample across all quarters). All models control for individual effects, age bin, and year fixed effects. In order to show how effects change with reference to pre-exposure use, model (2) additionally controls for outcome in first quarter after exposure, model (3) additionally controls for outcome in first and second quarter after exposure, model (4) additionally controls for outcome in first, second, and third quarter after exposure.**

Table S1. Estimated mean difference in mental health prescription use per quarter for up to four quarters after exposure for categories of working hours compared to not working

**Outcome**	**Mental health scripts per quarter**	**Mental health scripts per quarter**	**Mental health scripts per quarter**	**Mental health scripts per quarter**
Time period	First quarter after exposure<br>(1)<br>β [95% CI]	First quarter after exposure<br>(2)<br>β [95% CI]	Third quarter after exposure<br>(3)<br>β [95% CI]	Fourth quarter after exposure<br>(4)<br>β [95% CI]
unemployed (0 hours)	0.00000	0.00000	0.00000	0.00000
	[0.00000,0.00000]	[0.00000,0.00000]	[0.00000,0.00000]	[0.00000,0.00000]
(0-15)	-0.01721***	-0.03079***	-0.01934***	-0.02716***
	[-0.02250,-0.01192]	[-0.03614,-0.02544]	[-0.02572,-0.01295]	[-0.03475,-0.01956]
[15-30)	-0.04188***	-0.03170***	-0.03977***	-0.04706***
	[-0.04883,-0.03494]	[-0.03904,-0.02436]	[-0.04861,-0.03092]	[-0.05770,-0.03641]
30 +	-0.05174***	-0.04489***	-0.05940***	-0.08103***
	[-0.06136,-0.04212]	[-0.05511,-0.03468]	[-0.07233,-0.04647]	[-0.09657,-0.06549]
No. person-quarter observations	*6,669,765*	*6,495,245*	*6,320,426*	*6,145,914*

Notes: * Significant at 10%. ** Significant at 5%. * Significant at 1%. Standard errors are robust clustered at the individual level. Means provided for each outcome at the quarterly level (overall mean for the whole analytic sample across all quarters). All models control for individual effects, age bin, and year fixed effects. In order to show how effects change with reference to pre-exposure use, model (2) additionally controls for outcome in first quarter after exposure, model (3) additionally controls for outcome in first and second quarter after exposure, model (4) additionally controls for outcome in first, second, and third quarter after exposure.**
